# Targeting B-Raf inhibitor resistant melanoma with novel cell penetrating peptide disrupters of PDE8A – C-Raf

**DOI:** 10.1186/s12885-019-5489-4

**Published:** 2019-03-25

**Authors:** Connor M. Blair, Nicola M. Walsh, Bruce H. Littman, Frank W. Marcoux, George S. Baillie

**Affiliations:** 1 Institute of Cardiovascular and Medical Sciences, College of Medical, Veterinary and Life Sciences, University of Glasgow, Glasgow, UK; 2 Portage Glasgow Limited, Glasgow, UK; 3 Portage Pharmaceuticals Limited, Tortola, British Virgin Islands; 4 EyGen Inc, Wilmington, DE USA

**Keywords:** B-Raf, C-Raf, PDE8A, NRAS, PPL-008, CellPorter®, Disruption, Melanoma

## Abstract

**Background:**

Recent advances in the treatment of melanoma that involve immunotherapy and B-Raf inhibition have revolutionised cancer care for this disease. However, an un-met clinical need remains in B-Raf inhibitor resistant patients where first-generation B-Raf inhibitors provide only short-term disease control. In these cases, B-Raf inhibition leads to paradoxical activation of the C-Raf – MEK – ERK signalling pathway, followed by metastasis. PDE8A has been shown to directly interact with and modulate the cAMP microdomain in the vicinity of C-Raf. This interaction promotes C-Raf activation by attenuating the PKA-mediated inhibitory phosphorylation of the kinase.

**Methods:**

We have used a novel cell-penetrating peptide agent (PPL-008) that inhibits the PDE8A – C-Raf complex in a human malignant MM415 melanoma cell line and MM415 melanoma xenograft mouse model to investigate ERK MAP kinase signalling.

**Results:**

We have demonstrated that the PDE8A – C-Raf complex disruptor PPL-008 increased inhibitory C-Raf-S259 phosphorylation and significantly reduced phospho-ERK signalling. We have also discovered that the ability of PPL-008 to dampen ERK signalling can be used to counter B-Raf inhibitor-driven paradoxical activation of phospho-ERK in MM415 cells treated with PLX4032 (Vemurafenib). PPL-008 treatment also significantly retarded the growth of these cells. When applied to a MM415 melanoma xenograft mouse model, PPL-008C penetrated tumour tissue and significantly reduced phospho-ERK signalling in that domain.

**Conclusion:**

Our data suggests that the PDE8A-C-Raf complex is a promising therapeutic treatment for B-Raf inhibitor resistant melanoma.

**Electronic supplementary material:**

The online version of this article (10.1186/s12885-019-5489-4) contains supplementary material, which is available to authorized users.

## Background

Malignant melanoma, an aggressive and lethal form of skin cancer, leaves metastatic patients with a 15–20% chance of surviving 5 years with the disease [1]. Over 50% of melanoma patients carry a mutation in their BRAF gene, with the V600E (valine to glutamic acid) missense mutation being responsible for 80–90% of BRAF mutations [2–4]. B-Raf is a serine/threonine protein kinase that is part of the RAS – RAF – MEK – ERK signalling axis, involved in regulating many cellular processes including: differentiation, proliferation, survival and apoptosis [5]. This signalling pathway is believed to be crucial to melanoma progression, with the V600E mutation resulting in B-Raf protein conformational changes that constitutively activate B-Raf and downstream MEK – ERK signalling [5]. As a result, B-Raf-specific small molecule inhibitors (and eventually MEK inhibitors) were developed and found to dramatically improve patient prognosis, survival rate and lead to tumour regression through suppression of downstream ERK signalling [6–9]. Unexpectedly, B-Raf inhibitor resistance was developed in many patients through paradoxical activation of ERK; allowing the cancer to persist [10–14]. Pathway reactivation is believed to occur as a result of oncogenic mutations in a number of genes, including NRAS (20% of cases; Q61K/R/L most frequent) and KRAS (2% of cases) gain-of-function mutations [15] http://www.sanger.ac.uk/genetics/CGP/cosmic/ [16, 17]. As B-Raf preferentially heterodimerises to C-Raf (vs. other A, B or C-Raf homo/heterodimers), B-Raf inhibition results in a negative feedback mechanism that switches from B-Raf to C-Raf activation by Ras and subsequent tumour invasion and metastasis [18, 19]. In light of this, C-Raf has become a key therapeutic target for the development of new treatments able to suppress RAS-mediated tumour progression in B-Raf inhibitor resistant melanoma.

Previously, we demonstrated an important role for the cAMP degrading enzyme, PDE8A, in protecting C-Raf from PKA-mediated inhibition [20] (reviewed [21]). PDE8A, believed to be responsible for regulating basal cAMP fluctuations, was found to directly interact with C-Raf. The association of C-Raf with a PDE markedly inhibited the ability of local PKA pools to phosphorylate and inhibit the kinase, increasing the likelihood of C-Raf activation. Peptide mapping of the PDE8A-C-Raf interface allowed for the rational development of a cell penetrating peptide disrupter based on the C-Raf binding site on PDE8A [22, 23]. This disrupter was found to inhibit the PDE8A – C-Raf protein-protein interaction (PPI) and significantly increase C-Raf-S259 phosphorylation while concomitantly supressing phospho-ERK signalling. This concept was verified at an organismal level in both PDE8A knock out mice and a drosophila model, where basal ERK activation was attenuated compared to wild type [20].

Further verification of the PDE8A – C-Raf PPI inhibitor concept has been supplied by a recent study, which demonstrated that the disrupter was able to attenuate T-effector cell adhesion and migration in an auto-immune multiple sclerosis mouse model. The inhibition of T-effector cell function was a direct result of increased levels of inhibitory C-Raf-S259 phosphorylation and subsequent suppression of ERK activation [24]. The disrupter produced a more potent effect than highly-selective PDE8 enzyme inhibitors and highlighted a novel approach to targeting T-effector cells in inflammatory disorders. These observations convincingly demonstrate the disrupter’s ability to attenuate ERK activation through PDE8A – C-Raf disruption.

Further development of the PDE8A-C-Raf disrupter has allowed us to conjugate the peptide to the patented Cell Porter® (Portage Pharmaceuticals Limited), a cell penetrating peptide based on the human HOXD12 protein. The Cell Porter® platform has successfully driven PPL-003, an NF-kB inhibitor designed to treat inflammatory disorders including dry eye syndrome, to pre-clinical success [25–27]. We report data from the testing of our novel PDE8A – C-Raf disrupter/HOXD12 conjugate (from here on, named PPL-008) in MM415, a B-Raf inhibitor resistant malignant melanoma cell line (MM415; BRAF wt, KRAS wt, NRAS Q61L) and in an MM415 melanoma murine xenograft model. PPL-008 was efficacious in the attenuation of ERK signalling in both cases and suggests that the PDE8A – C-Raf complex is a promising therapeutic target for B-Raf inhibitor resistant melanoma.

## Methods

All antibodies and chemical treatments are collated in Additional file 1: Table S1.

### Animals

Generation of the MM415 melanoma murine xenograft model, and in vivo treatment was carried out by MI Bioresearch (Michigan, USA). All protocols involving animals used were approved by the Institutional Animal Care and Use Committee of the University of Washington in accordance with the National Institutes of Health. In vivo 5–6 week old female NSG – immunodeficient mice (Jackson Laboratory) were subcutaneously injected with 3.3e+ 8 MM415 malignant melanoma cells, at the SC – axilla (high), and tumours were allowed to grow for 30 days (~ 200 – 400mm^3^). Mice were intraperitoneally injected at the site of tumour with PPL-008 peptide drug dissolved in a 5% dextrose – water solution at either 25 mg/kg or 100 mg/kg. Mice were euthanised via CO_2_ inhalation (MI Bioresearch – AALAC accredited laboratory) and the tumours were harvested at varying time points post-treatment: 30 min, 1 h, 2 h, 4 h, 8 h, 12 h. Tumours were frozen down at − 80 **°**C and sent to Baillie lab for preparation into lysates for follow-up western blot analyses.

### MM415 cell culture and drug treatments

Both cell lines used in this study, A375 and MM415, were purchased from Sigma-Aldrich. A375 (BRAF V600E) and MM415 (BRAF wild-type, KRAS wild-type, NRAS Q61L) are human malignant melanoma epithelial skin cell lines. Cells were cultured with RPMI 1640 medium, supplemented with 10% fetal bovine serum (FBS, *v*/v), 1% L-glutamine (v/v), 1% penicillin-streptomycin (v/v) (all Sigma-Aldrich) and incubated at 37 **°**C, 5% CO_2_ and 95% humidity. Cells were split at ~ 80% confluency, using 0.05% trypsin-EDTA, 1:5. Cells were tested regularly for mycoplasma contamination.

The original PDE8A – C-Raf disrupter, and its scrambled isoform, were synthesised with a C-terminal stearic acid group [CH3(CH2)16COOH] (GenScript) [20]. PPL-008 (i.e. PDE8A – C-Raf disrupter, without stearic acid) was synthesised with Cell Porter® conjugated to the C or N-terminus via either thioester or disulphide bonds. All peptides were dissolved to the appropriate concentration in DMSO for in vitro experimentation. PLX4032 (Vemurafenib) was dissolved in DMSO to a final concentration of 1 μM (Sellekchem). Peptides were added to cells for 2 h, and PLX for 1 h, before cells were harvested. In cases where co-treatments were administered, cells were first treated with peptides for 2 h, followed by 1 h PLX.

### Western blotting

MM415 and A375 cells harvested from in vitro experiments were lysed in 3 T3 lysis buffer, whilst MM415 melanoma murine xenograft tissue was homogenised and lysed in 1X RIPA buffer (both supplemented with protease cocktail inhibitor tablets (Roche)). Soluble fraction of lysate was resolved via SDS/PAGE using 4–12% Bis-Tris gels (NuPAGE). Proteins were transferred at 30 V for 1 h onto 0.45 μm nitrocellulose membrane (Protran) and blocked for 1 h in 5% non-fat dry milk solution (Marvel, *w*/*v*) in 1x TBS-T (20 mM Tris-Cl pH 7.6, 150 mM NaCl, 0.1% Tween-20). Blocked membranes were incubated in primary antibody (diluted in 1x TBS-T, 1% marvel) overnight at 4 **°**C. Membranes were washed three times in 1x TBS-T before membranes were incubated in secondary antibody (diluted in 1x TBS-T, 1% marvel) for 1 h at room temperature. Membranes were washed a final three times in 1x TBS-T and fluorescent intensity of Li-Cor secondary antibody was measured using a Li-Cor Odyssey scanner.

### xCELLigence: Measuring cell proliferation

Real-Time cellular growth analyses of MM415 cells, using the xCELLigence platform (Roche Applied Science), allowed for the label-free measurement of cell proliferation. 96 well E-plates, containing gold microelectrode sensors on the bottom of the plate, were used to measure cellular impedance inside each well as per manufacturer’s instructions. Cellular impedance measurements were translated into ‘cell index’, an arbitrary measurement that increases as MM415 cells adhere and spread-out/grow (and vice versa), giving quantitative information on cell proliferation and viability that were analysed using RTCA software (Roche). All protocols carried out using the xCELLigence platform were based on previous Baillie lab publications [20, 28–32]. Following MM415 cell adhesion, cells were treated with one of the peptide disrupters for 2 h, followed by PLX (1 μM). The slope (i.e. rate of cell proliferation/growth) was measured based on the normalised cell index from the point in which treatments were administered, until the response had plateaued appropriately.

### Statistical analyses

Results from western blot analyses are represented as mean ± SEM (*n* ≥ 3). Results from xCELLigence cell proliferation assay are represented as mean ± STDEV (*n* ≥ 3). *P* < 0.05 indicates data are significant, with significance determined via unpaired t-test using GraphPad Prism software.

## Results

### PPL-008 attenuates paradoxical activation of pERK signalling and MM415 cell proliferation

**Fig. 1:**
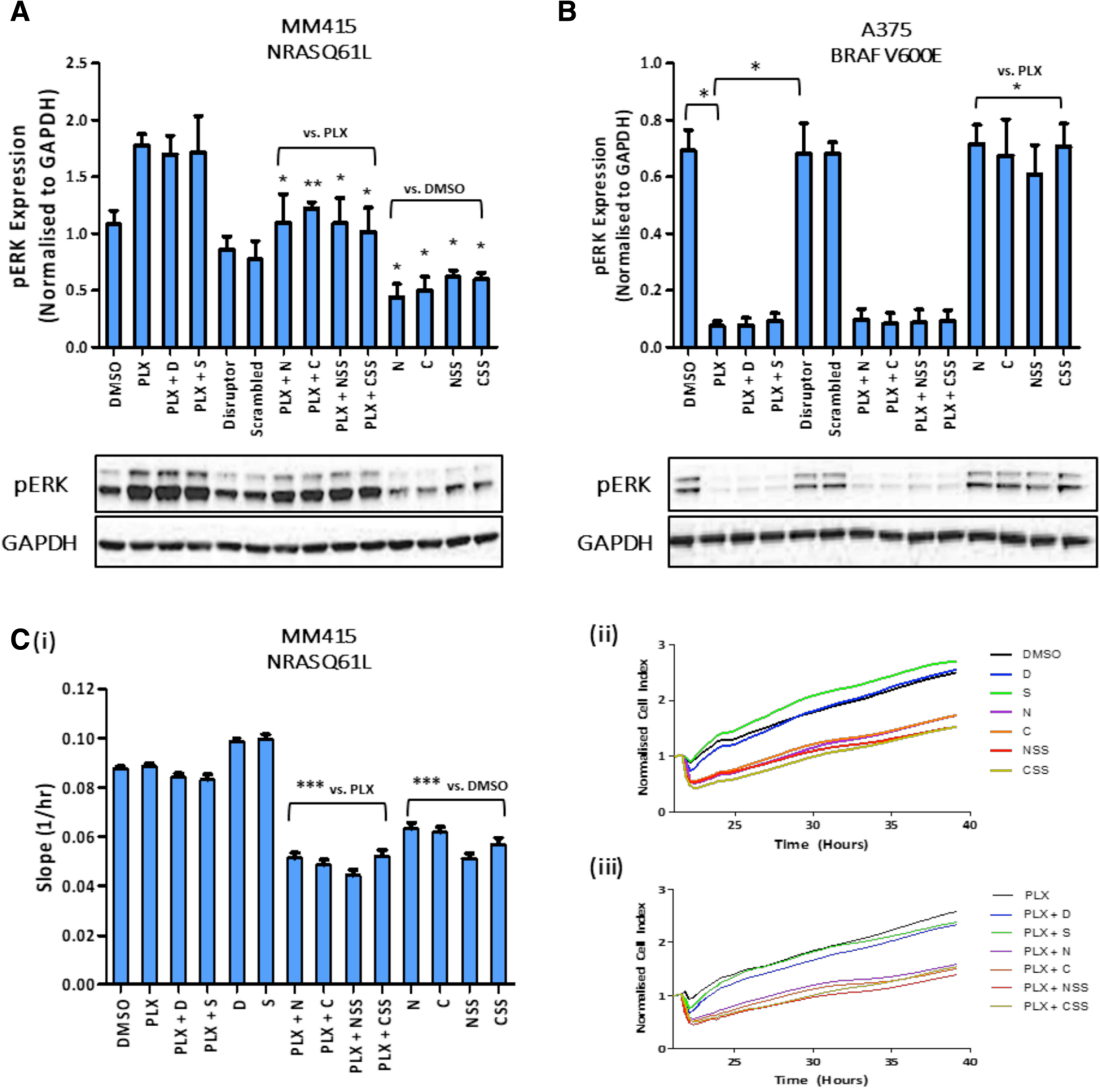
Effects of PPL008 conjugates on pERK levels and rate of cell proliferation. Normalised phospho-ERK (mean ± SEM) following treatment with DMSO (lane 1), PLX4032 B-Raf inhibitor (lane 2), PPL008 conjugates (lanes 11–14) or PLX co-treatments with PDE8A – C-Raf peptide disrupters (original stearylated ‘disrupter’ lane 3, or scrambled control, lane 4) or PPL-008 conjugates (lanes 7–10) in: **a** MM415 (NRAS Q61L) and (**b**) A375 (BRAF V600E) human malignant melanoma cell lines. Respective pERK and GAPDH immunoblot examples shown below (*N* ≥ 3, * P < 0.05, ** P < 0.01). **c** Real-time cell analyses (xCELLigence platform) of MM415 cell proliferation following treatments described above. Treatments occurred at 21 h and the slope of normalised cell index (mean ± STDEV) was measured between 21 and 39 h, with (ii) representing DMSO vs. peptide disrupter treatments only (10 μM) and (iii) representing PLX (1 μM) vs. co-treatments of peptide disrupters and PLX4032 (*n* = 3, *** *P* < 0.001)*. D, stearylated disrupter; S, stearylated scrambled; N, PPL-008 N; C, PPL-008C; NSS, PPL-008NSS; CSS, PPL-008CSS*

Successful inhibition of the Raf – MEK – ERK signalling axis has previously been shown, using a PDE8A – C-Raf peptide disrupter based on the C-Raf binding site on PDE8A [20]. Here, we conjugated the Cell Porter® onto the C or N-terminal of the disrupter via either thioester (C or N giving PPL-008C or PPL-008 N) or disulphide bonds (CSS or NSS giving PPL-008CSS or PPL-008NSS). We used the peptide conjugates to treat the B-Raf inhibitor resistant MM415 (human malignant melanoma, BRAF wt, KRAS wt, NRAS Q61L) cell line. To determine if PPL-008 conjugates (10 μM) could suppress phospho-ERK signalling in MM415 cells, pERK levels were determined via western blot (*pERK expression normalised to GAPDH, mean ± SEM, n = 4,* Fig. 1). MM415 B-Raf inhibitor treatment (PLX4032, 1 μM) clearly induced a paradoxical activation of ERK and this was significantly reduced following treatment with all the analogues (PPL-008 N, PPL-008C, PPL-008NSS and PPL-008CSS) (** P < 0.01 or * P < 0.05; Fig. 1a*, lanes 7–10 inclusive*). As expected, pERK was significantly reduced in the human A375 malignant melanoma cell line (BRAF V600E) following PLX treatment (1 μM) (Fig. 1b, *lanes 2,3,4*) with PPL-008 analogues providing no ERK inhibition as a mono-treatment (Fig. 1b, *lanes 11–14 inclusive*) or further ERK inhibition as a co-treatment with PLX (Fig. 1b*, lanes 7–10 inclusive*). This data reinforces how effective B-Raf inhibition can be in treating BRAF V600E mutant melanoma [6, 7]. As B-Raf inhibition sufficiently suppressed pERK expression in A375 cells, and as MM415 cells were resistant to PLX (resembling the clinical phenotype of interest), MM415 cells were used for the remainder of the study.

To assess the ability of PPL-008 conjugates to inhibit cell growth, real-time measurements of MM415 cellular impedance was recorded on the xCELLigence platform as an indicator of cellular proliferation *(slope (1/h), mean ± STD, n = 3)*. All PPL-008-conjugates (10 μM) significantly slowed cell proliferation in PLX treated MM415 cells (* P < 0.05, Fig. 1c*, lanes 7–14 inclusive*)*.* Our data suggests treatment (10 μM) with each of the PPL-008 analogues suppressed both pERK and cell growth compared with DMSO treated control (Fig. 1a and c)*,* indicating PPL-008 has potential as an effective therapy in this context. Surprisingly, PPL-008C and PPL-008CSS mono-treatments significantly attenuated A375 growth (Additional file 2: Figure S1) without affecting the phospho-ERK profile (Fig. 1b, *lanes 11–14 inclusive*). It is noteworthy that, the original stearylated PDE8A – C-Raf disrupter caused no significant reduction in pERK signalling *(‘D’,* Fig. 1a*, lane 5*) or cell proliferation (*‘D’,* Fig. 1c*, lane 5)* in MM415, similar to its scrambled control (*‘S’,* Fig. 1a*, lane 6 and* Fig. 1c*, lane 6*), suggesting the stearic acid group was insufficient in facilitating cell-penetration in MM415 cells. As all PPL-008 conjugates attenuated both pERK expression and cell proliferation, this indicates that Cell Porter® greatly improves intracellular delivery of PPL-008 conjugates compared with the original disrupter’s stearate group.

### PPL-008C / N inhibits pERK expression and MM415 cell proliferation over multiple doses

**Fig. 2:**
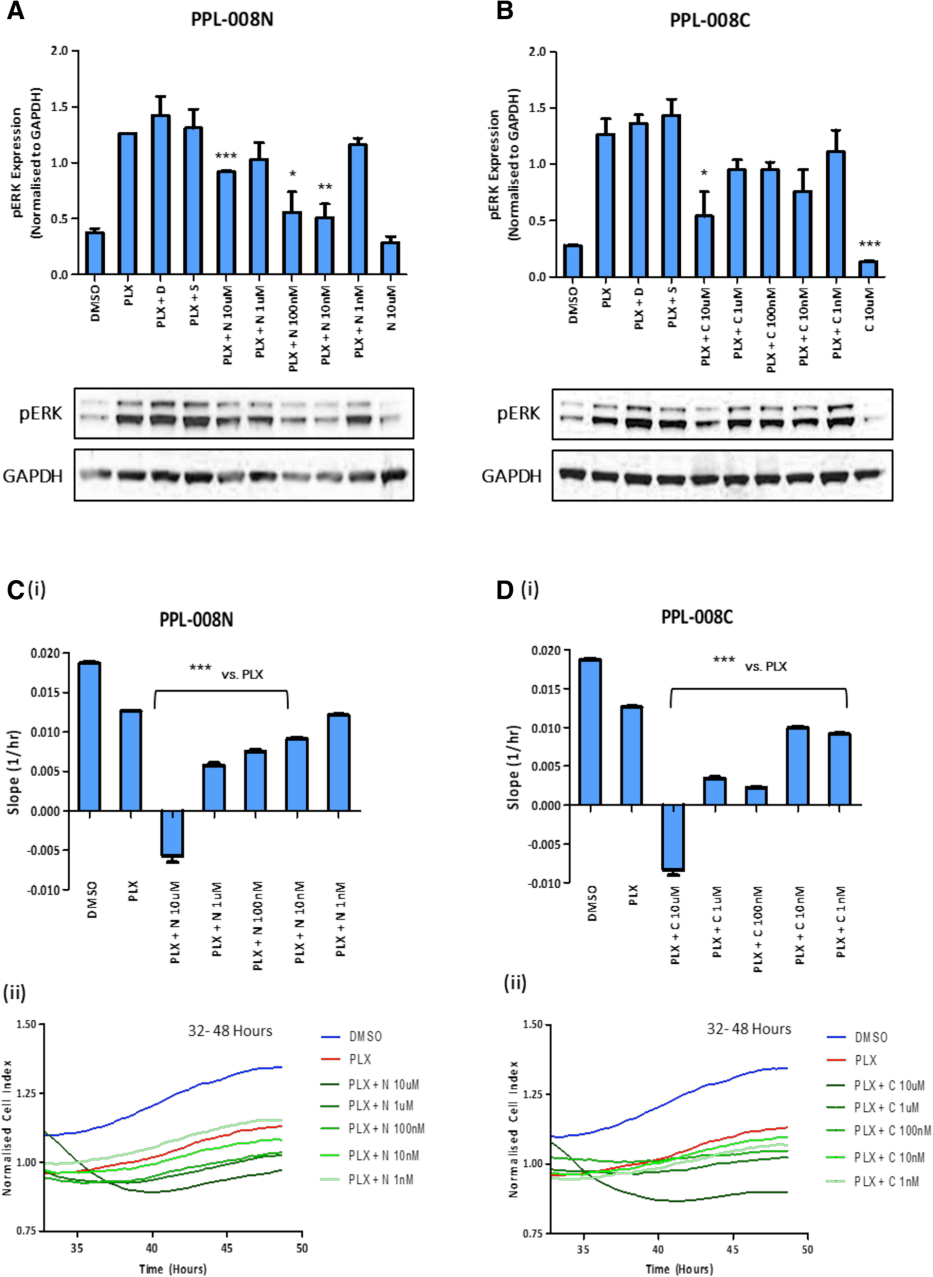
PPL008-C/N dose response in MM415 (NRAS Q61L) human malignant melanoma cell line. **a** & **b**: Normalised pERK expression (mean ± SEM) in MM415 cells following dose response co-treatment with PLX4032 (1 μM) and PPL-008 N or PPL-008C (1 nM – 10 μM). Respective pERK and GAPDH immunoblot examples shown below. (N = 3, * P < 0.05, ** P < 0.01, *** P < 0.001), DMSO vs. *PPL008 only, PLX* vs. *PLX + PPL008).* **c** & **d** (i) Real-time cell analyses (xCELLigence platform) of MM415 cell proliferation following dose response co-treatments with PPL-008 N or PPL-008C (1 nM - 10 μM) and PLX4032 (1 μM). Treatments occurred at 32 h and the slope of normalised cell index (mean ± STDEV) was measured between 32 and 48 h (*n* = 3, *** *P* < 0.001). **c** & **d** (ii) Representative traces of normalised cell index of each treatment shown below. *N, PPL-008 N; C, PPL-008C*

MM415 cells were co-treated with PLX (1 μM), following pre-treatment with a dose range of PPL-008C or PPL-008 N (1 nM – 10 μM). The levels of pERK and cell proliferation rates determined as before (Fig. 2). The levels of pERK triggered by PLX (Fig. 2a and b*, lane 1* vs *lane 2*) were reduced at all concentrations following PPL-008 N treatment, with the higher [10 μM] dose causing the most significant reduction (*** P < 0.001, Fig. 2a). This effect was recapitulated in the xCELLigence cell proliferation assay, where PPL-008 N reduced the rate of cell proliferation at all concentrations; most significantly at 10 μM (*** P < 0.001, Fig. 2c *(i and ii)*). In addition, PPL-008C-conjugate reduced MM415 pERK levels and rate of cell proliferation at all concentrations, with [10 μM] producing the most significant inhibition (*** P < 0.001, Fig. 2b and d *(i and ii)*).

### In vivo PPL-008C suppression of pERK in an MM415 melanoma murine xenograft model

**Fig. 3:**
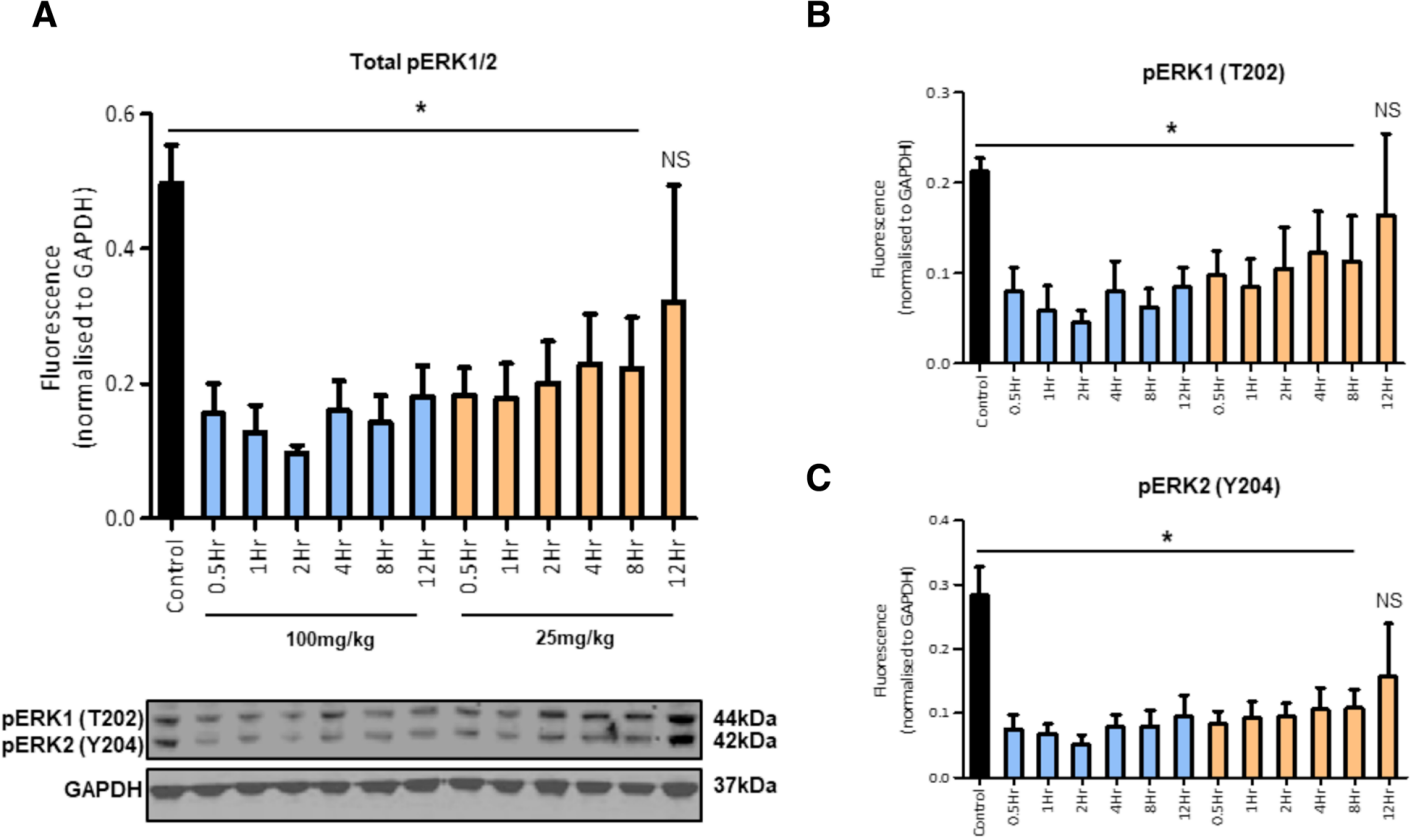
In vivo suppression of phospho-ERK signalling in an MM415 murine xenograft model. **a** Normalised total pERK1/2, **b** pERK1 (T202, 44 kDa) and (**c**) pERK2 (Y204, 42 kDa) levels (mean ± SEM) in MM415 (Q61L) tumour xenografts from NSG immuno-deficient mice following PPL-008C treatment, at multiple time points, with either 25 mg/kg or 100 mg/kg doses (control *N* = 3, treated *N* = 4, * *P < 0.05*). Control mice were treated with a 5% dextrose in dH_2_O solution and PPL-008C was administered via subcutaneous injection at the site of tumour. Representative pERK1/2 and GAPDH immunoblot examples shown below (**a**)

**Fig. 4:**
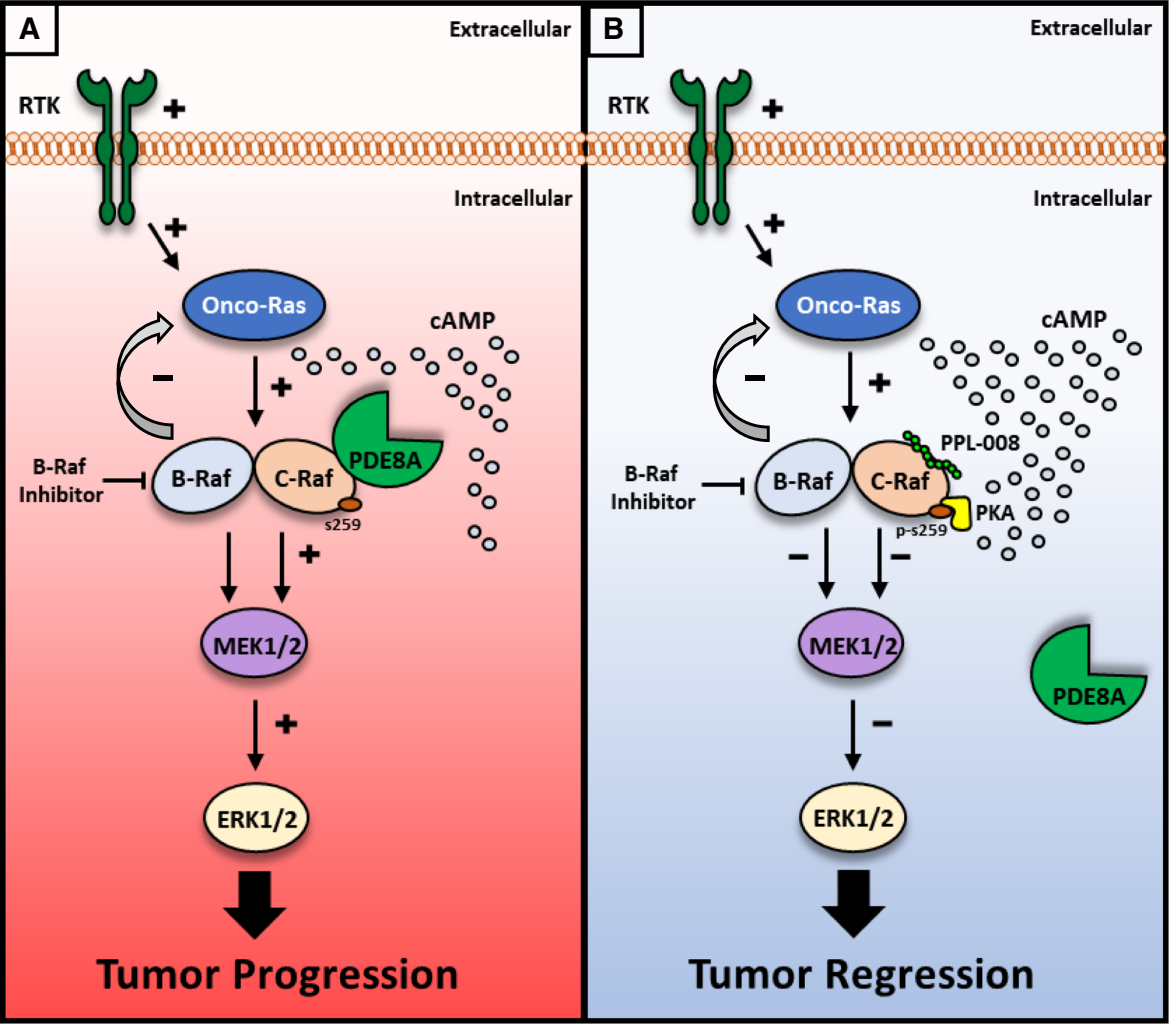
Dual inhibition of B-Raf and C-Raf inhibits melanoma tumour progression. **a** B-Raf inhibition leads to the Ras negative feedback mechanism switching to C-Raf driven tumourigenesis via potentiation of the Raf/MEK/ERK signalling axis. **b** PPL-008 (PDE8A – C-Raf disrupter peptide) binds to C-Raf, preventing PDE8A localisation within the C-Raf cAMP microdomain and exposing serine 259 – C-Raf to inhibitory phosphorylation by PKA. Co-treatment with B-Raf inhibitor and PPL-008 blocks onco-Ras driven tumour progression via inhibition of the Raf/MEK/ERK axis

Preliminary in vivo investigation of the effects of PPL-008C were carried out in an immuno-deficient NSG – MM415 melanoma murine xenograft model. PPL-008C was chosen as the lead peptide disrupter due to its consistency in attenuating pERK signalling and cell proliferation as both a single treatment and co-treatment with PLX (Figs. 2, 3 and 4). PPL-008C was administered subcutaneously at the site of the tumour as a single treatment. Tumours were removed at varying time points post-treatment and pERK expression was assessed via western blot (*N ≥ 3,* Fig. 3).

PPL-008C significantly suppressed pERK1, pERK2 and total pERK levels over all time-points in the time course and at both doses (25 mg/kg and 100 mg/kg), excluding the 25 mg/kg – 12Hr treatment (* P < 0.05, Fig. 3). This shows that a single PPL-008C treatment can attenuate Raf – MEK – ERK signalling relatively quickly (within 30 min) and can maintain this inhibition for at least 12 h at higher concentrations (100 mg/kg, Fig. 3). Maximal pERK inhibition occurred 2 h post-treatment with 100 mg/kg PPL-008C.

## Discussion

Melanoma is the most aggressive form of skin cancer, with a wide range of treatments currently available and many more at pre-clinical and clinical phases [8, 22, 23]. First line B-Raf inhibitors are capable of managing the majority of melanoma patients that express the BRAF V600E mutation [23]. However, in patients expressing wildtype BRAF and NRAS or KRAS gain-of-function mutations, B-Raf inhibitors become ineffective and tumours persist – warranting the development of novel effective treatments [10–15] http://www.sanger.ac.uk/genetics/CGP/cosmic/ [16, 17] (Fig. 4). We have identified the PDE8A – C-Raf complex as a point of cross-talk between the MAP kinase signalling and cAMP signalling systems, that can be manipulated by a disrupter peptide to promote the inhibition of C-Raf via increased S259 phosphorylation [20, 21, 24] (Fig. 4). This action can counteract C-Raf driven paradoxical activation of ERK in B-Raf inhibitor resistant melanoma cell lines resulting in a retardation of cell proliferation (Figs. 1 and 2). Our specific approach of inhibiting C-Raf by targeting its binding to anchoring proteins rather than kinase activity is, to our knowledge, novel for this kinase and we aim to displace only a small percentage of total C-Raf that is in complex with PDE8A. Protein-protein interactions (PPIs) are increasingly being regarded as tractable molecular targets for the development of therapeutics, and peptides that mimic docking sites with protein complexes are often the ideal scaffold starting point for such agents [33].

The concept of developing protein-protein interaction inhibitors to treat melanoma, however, has previously been investigated with small molecule PPI inhibitors of the complex between bromodomain-containing protein 4 (BRD4) and acetylated histone having been developed [34]. Chromatin immunoprecipitation (ChIP) analysis highlighted the ability of these compounds to antagonise the interaction between BRD4 and chromatin at the MYC promoter in melanoma cells to effect the down regulation of oncogenic c-myc. This approach also demonstrated potent anti-proliferative in vivo activity in A375 xenografts. Another PPI inhibitor that targets melanoma growth, via the dual targeting of tumour and endothelial cells, is C4 [35]. This molecule targets the C-terminus of Focal Adhesion Kinases (FAK) to interdict the kinase’s interaction with VEGF-receptor 3 and subsequently reduce vascularisation of B-RAF V600E xenograft tumour tissue to limit blood flow [36]. The inhibition of PPIs by small molecules as a therapeutic strategy is also being evaluated for other cancers. High content compound screening has identified Androgen receptor – Transcription Intermediary Factor 2 (TIF2) disrupters for prostate cancer [37], menin-mixed lineage leukemia 1 (MLL1) disrupters for leukemia [38, 39], B-cell lymphoma 6 (BCL6) – BCL6 corepressor (BCOR) disrupters for treatment of diffuse large B-cell lymphomas [40], Rictor – mTOR blockers for glioblastoma [41] and P53 – MDM2 inhibitors for a range of treatment resistant cancer types [42, 43].

However, although the above cases relate to small molecule inhibitors, instances of peptide PPI inhibitors as novel anti-cancer agents are also beginning to emerge. Recently, for example, cell permeable peptides have been developed against the NEMO – IκB kinase complex for the treatment of cisplatin-resistant ovarian cancer [44], stapled peptide disrupters that unhook β-Catenin from transcription factors have been produced as novel colon cancer agents [45] and colon cancer has also been the target of adenomatous polyposis coli (APC) – Asef disrupters [46] that inhibit the migration and invasion of colon cancer cell lines.

Cell delivery of therapeutic peptides in the cancer sphere has been undertaken by a variety of different routes involving liposomes [47], nanoparticles [48] and short cell-penetrating sequences (reviewed [49]). Our peptide is conjugated to Cell Porter® (Portage Pharmaceuticals Limited), a patented cell penetrating peptide based on the human HOXD12 protein [25–27]. Previously we have utilised stearate groups to good effect to deliver PPI inhibitor peptides into cellular [50, 51] and animal models of disease [52], however on this occasion the stearylated peptides had little effect on the levels of phospho-ERK in MM415 cells (Fig. 1a and c). Evidently, the effectiveness of peptide delivery systems is context specific and our data shows that CellPorter® has directed intracellular delivery of a novel C-Raf – PDE8A peptide disrupter leading to significant suppression of paradoxical ERK activation in a clinically relevant B-Raf inhibitor resistant human melanoma cell line and an apt xenograft model of the disease.

## Conclusion

PPL-008 conjugates represent potential starting points for the development of co-therapies for resistant melanoma to be administered with an appropriate B-Raf inhibitor in order to overcome B-Raf inhibitor resistance and attenuate ERK activation in melanocytes.

## Additional files

Additional file 1: **Table S1.** Chemicals and Antibodies used in study. (DOCX 67 kb)

Additional file 2: A375 growth data. Single-treatment of 10 μM PPL-008C and PPL-008CSS significantly attenuated A375 cell (V700E malignant melanoma cell line) growth although pERK levels are unaffected by these treatments (see Fig. 1.). (PPTX 118 kb)

## Abbreviations

cAMP: cyclic adenosine monophosphate
CellPorter®: HOXD12 cell-penetrating peptide
C-Raf-S259: Inhibitory C-Raf serine residue 259
ERK: Mitogen-activated protein kinase
MEK: Mitogen-activated protein kinase of ERK
PDE8A: Phosphodiesterase 8A
PLX/Vemurafenib: Plexxikon developed B-Raf enzyme inhibitor
PPL008: PDE8A – C-Raf peptide disrupter
RAF: Serine/threonine protein kinase

## References

[1] Melanoma UK Statistics. http://www.melanomauk.org.uk/about_melanoma/statistics/ Available 23 Apr 2017.

[2] Davies H, Bignell GR, Cox C, Stephens P, Edkins S, Clegg S (2002). Mutations of the BRAF gene in human cancer. Nature..

[3] Shinozaki M, Fujimoto A, Morton DL, Hoon DSB (2004). Incidence of BRAF oncogene mutation and clinical relevance for primary cutaneous melanomas. Clin Cancer Res.

[4] Lovly C, Pao W, Sosman J. BRAF c.1799T>a (V600E) mutation in melanoma. My Cancer. 2015; https://www.mycancergenome.org/content/disease/melanoma/braf/54/ Available 29 Apr 2017.

[5] McCubrey JA, Steelman LS, Chappell WH, Abrams SL, Wong EWT, Chang F (2007). Roles of the Raf/MEK/ERK pathway in cell growth, malignant transformation and drug resistance. Biochimica et Biophysica Acta - Molecular Cell Research.

[6] Flaherty KT, Puzanov I, Kim KB, Ribas A, McArthur GA, Sosman JA (2010). Inhibition of mutated, activated BRAF in metastatic melanoma. N Engl J Med.

[7] Joseph EW, Pratilas CA, Poulikakos PI, Tadi M, Wang W, Taylor BS (2010). The RAF inhibitor PLX4032 inhibits ERK signaling and tumor cell proliferation in a V600E BRAF-selective manner. Proc Natl Acad Sci.

[8] Domingues B, Lopes JM, Soares P, Pópulo H (2018). Melanoma treatment in review. ImmunoTargets Ther.

[9] Griffin M, Scotto D, Josephs DH, Mele S, Crescioli S, Bax HJ (2017). BRAF inhibitors : resistance and the promise of combination treatments for melanoma. Oncotarget..

[10] Halaban R, Zhang W, Bacchiocchi A, Cheng E, Parisi F, Ariyan S (2010). PLX4032, a selective BRAFV600E kinase inhibitor, activates the ERK pathway and enhances cell migration and proliferation of BRAFWT melanoma cells. Pigment Cell Melanoma Res.

[11] Oh YT, Deng J, Yue P, Sun SY (2016). Paradoxical activation of MEK/ERK signaling induced by B-Raf inhibition enhances DR5 expression and DR5 activation-induced apoptosis in Ras-mutant cancer cells. Sci Rep.

[12] Gibney GT, Messina JL, Fedorenko IV, Sondak VK, Smalley KSM (2013). Paradoxical oncogenesis-the long-term effects of BRAF inhibition in melanoma. Nat Rev Clin Oncol.

[13] Poulikakos PI, Zhang C, Bollag G, Shokat KM, Rosen N (2010). RAF inhibitors transactivate RAF dimers and ERK signalling in cells with wild-type BRAF. Nature..

[14] Heidorn SJ, Milagre C, Whittaker S, Nourry A, Niculescu-Duvas I, Dhomen N (2010). Kinase-dead BRAF and oncogenic RAS cooperate to drive tumor progression through CRAF. Cell..

[15] Sanchez-Laorden B, Viros A, Girotti MR, Pedersen M, Saturno G, Zambon A, et al. BRAF inhibitors induce metastasis in RAS mutant or inhibitor-resistant melanoma cells by reactivating MEK and ERK signaling. Sci Signal. 2014;7(318). 10.1126/scisignal.2004815 24667377

[16] Dumaz N, Hayward R, Martin J, Ogilvie L, Hedley D, Curtin JA (2006). In melanoma, RAS mutations are accompanied by switching signaling from BRAF to CRAF and disrupted cyclic AMP signaling. Cancer Res.

[17] Marquette A, André J, Bagot M, Bensussan A, Dumaz N (2011). ERK and PDE4 cooperate to induce RAF isoform switching in melanoma. Nat Struct Mol Biol.

[18] Rushworth LK, Hindley AD, O’Neill E, Kolch W (2006). Regulation and role of Raf-1/B-Raf Heterodimerization. Mol Cell Biol.

[19] Durrant DE, Morrison DK (2018). Targeting the Raf kinases in human cancer: the Raf dimer dilemma. Vol. 118. Br J Cancer.

[20] Brown KM, Day JP, Huston E, Zimmermann B, Hampel K, Christian F (2013). Phosphodiesterase-8A binds to and regulates Raf-1 kinase. Proc Natl Acad Sci.

[21] Maurice DH (2013). PDE8A runs interference to limit PKA inhibition of Raf-1. Proc Natl Acad Sci.

[22] Min DH, Mrksich M. Peptide arrays: towards routine implementation. Curr Opin Chem Biol. 2004;8:554–8. 10.1016/j.cbpa.2004.08.007 15450500

[23] Katz C, Levy-Beladev L, Rotem-Bamberger S, Rito T, Rüdiger SGD, Friedler A (2011). Studying protein–protein interactions using peptide arrays. Chem Soc Rev.

[24] Basole CP, Nguyen RK, Lamothe K, Vang A, Clark R, Baillie GS (2017). PDE8 controls CD4+T cell motility through the PDE8A-Raf-1 kinase signaling complex. Cell Signal.

[25] Littman BH, Marcoux FW, Jamison JA (2016). Efficacy of PPL-003 and the role of NFκB activation in a rat model of dry eye disease. Invest Ophthalmol Vis Sci.

[26] Rosenbaum JT, Littman BH, Marcoux FW, Jamison JA (2016). Efficacy of PPL-003 and inhibition of NFκB activation in a rabbit mycobacterial antigen-induced uveitis model. Invest Ophthalmol Vis Sci.

[27] Littman BH, Jamison JA, Ochoa R (2017). Rabbit safety of topical PPL-003: a cell penetrating peptide inhibitor of NFkB for dry eye disease. Invest Ophthalmol Vis Sci.

[28] Anthony DF, Sin YY, Vadrevu S, Advant N, Day JP, Byrne AM (2011). β-Arrestin 1 inhibits the GTPase-activating protein function of ARHGAP21, promoting activation of RhoA following angiotensin II type 1A receptor stimulation. Mol Cell Biol.

[29] Henderson DJP, Byrne A, Dulla K, Jenster G, Hoffmann R, Baillie GS (2014). The cAMP phosphodiesterase-4D7 (PDE4D7) is downregulated in androgen-independent prostate cancer cells and mediates proliferation by compartmentalising cAMP at the plasma membrane of VCaP prostate cancer cells. Br J Cancer.

[30] Sin YY, Martin TP, Wills L, Currie S, Baillie GS (2015). Small heat shock protein 20 (Hsp20) facilitates nuclear import of protein kinase D 1 (PKD1) during cardiac hypertrophy. Cell Commun Signal.

[31] Cameron RT, Whiteley E, Day JP, Parachikova AI, Baillie GS (2017). Selective inhibition of phosphodiesterases 4, 5 and 9 induces HSP20 phosphorylation and attenuates amyloid beta 1–42-mediated cytotoxicity. FEBS Open Bio..

[32] Day JP, Whiteley E, Freeley M, Long A, Malacrida B, Kiely P, et al. RAB40C regulates RACK1 stability via the ubiquitin – proteasome system. Futur Scii OA. 2018;4(7). 10.4155/fsoa-2018-0022 PMC6088270 30112187

[33] Pelay-Gimeno M, Glas A, Koch O, Grossmann TN (2015). Structure-based Design of Inhibitors of protein-protein interactions: mimicking peptide binding epitopes. Angew Chem Int Ed Engl..

[34] Zhong HJ, Lu L, Leung KH, Wong CCL, Peng C, Yan SC, Ma DL, Cai Z, David Wang HM, Leung CH (2015). An iridium (iii)-based irreversible protein-protein interaction inhibitor of BRD4 as a potent anticancer agent. Chem Sci.

[35] Kurenova EV, Hunt DL, He D, Magis AT, Ostrov DA, Cance WG (2009). Small molecule chloropyramine hydrochloride (C4) targets the binding site of focal adhesion kinase and vascular endothelial growth factor receptor 3 and suppresses breast cancer growth in vivo. J Med Chem.

[36] Kurenova E, Ucar D, Liao J, Yemma M, Gogate P, Bshara W, Sunar U, Seshadri M, Hochwald SN, Cance WG (2014). A FAK scaffold inhibitor disrupts FAK and VEGFR-3 signaling and blocks melanoma growth by targeting both tumor and endothelial cells. Cell Cycle.

[37] Fancher AT, Hua Y, Camarco DP, Close DA, Strock CJ, Johnston PA (2018). High-content screening campaign to identify compounds that inhibit or disrupt androgen receptor-transcriptional intermediary factor 2 protein-protein interactions for the treatment of prostate Cancer. Assay Drug Dev Technol.

[38] Xu S, Aguilar A, Xu T, Zheng K, Huang L, Stuckey J, Chinnaswamy K, Bernard D, Fernandez-Salas E, Liu L, Wang M, McEachern D, Przybranowski S, Foster C, Wang S (2018). Design of the First-in-Class, highly potent irreversible inhibitor targeting the Menin-MLL protein-protein interaction. Angew Chem Int Ed Engl.

[39] Borkin D, Klossowski S, Pollock J, Miao H, Linhares BM, Kempinska K, Jin Z, Purohit T, Wen B, He M, Sun D, Cierpicki T, Grembecka J (2018). Complexity of blocking bivalent protein-protein interactions: development of a highly potent inhibitor of the Menin-mixed-lineage leukemia interaction. J Med Chem.

[40] Sameshima T, Yamamoto T, Sano O, Sogabe S, Igaki S, Sakamoto K, Ida K, Gotou M, Imaeda Y, Sakamoto J, Miyahisa I (2018). Discovery of an irreversible and cell-active BCL6 inhibitor selectively targeting Cys53 located at the protein-protein interaction Interface. Biochemistry..

[41] Benavides-Serrato A, Lee J, Holmes B, Landon KA, Bashir T, Jung ME, Lichtenstein A, Gera J (2017). Specific blockade of Rictor-mTOR association inhibits mTORC2 activity and is cytotoxic in glioblastoma. PLoS One.

[42] Clark RC, Lee SY, Searcey M, Boger DL (2009). The isolation, total synthesis and structure elucidation of chlorofusin, a natural product inhibitor of the p53-mDM2 protein-protein interaction. Nat Prod Rep.

[43] Okamoto T (2008). Molecular docking analysis of the protein-protein interaction between RelA-associated inhibitor and tumor suppressor protein p53 and its inhibitory effect on p53 action. Cancer Sci.

[44] Rhodes CA, Dougherty PG, Cooper JK, Qian Z, Lindert S, Wang QE, Pei D (2018). Cell-permeable bicyclic peptidyl inhibitors against NEMO-IkappaB kinase interaction directly from a combinatorial library. J Am Chem Soc.

[45] Dietrich L, Rathmer B, Ewan K, Bange T, Heinrichs S, Dale TC, Schade D, Grossmann TN (2017). Cell permeable stapled peptide inhibitor of Wnt signaling that targets beta-catenin protein-protein interactions. Cell Chem Biol.

[46] Jiang H, Deng R, Yang X, Shang J, Lu S, Zhao Y, Song K, Liu X, Zhang Q, Chen Y, Chinn YE, Wu G, Li J, Chen G, Yu J, Zhang J (2017). Peptidomimetic inhibitors of APC-Asef interaction block colorectal cancer migration. Nat Chem Biol.

[47] Zhang Y, Zhang L, Hu Y, Jiang K, Li Z, Lin YZ, Wei G, Lu W (2018). Cell-permeable NF-kappaB inhibitor-conjugated liposomes for treatment of glioma. J Control Release.

[48] He X, Chen X, Liu L, Zhang Y, Lu Y, Zhang Y, Chen Q, Ruan C, Guo Q, Li C, Sun T, Jiang C (2018). Sequentially triggered nanoparticles with tumor penetration and intelligent drug release for pancreatic Cancer therapy. Adv Sci (Weinh).

[49] Farkhani SM, Valizadeh A, Karami H, Mohammadi S, Sohrabi N, Badrzadeh F (2014). Cell penetrating peptides: efficient vectors for delivery of nanoparticles, nanocarriers, therapeutic and diagnostic molecules. Peptides..

[50] Yalla K, Elliott C, Day JP, Findlay J, Barratt S, Hughes ZA, Wilson L, Whiteley E, Popiolek M, Li Y, Dunlop J, Killick R, Adams DR, Brandon NJ, Houslay MD, Hao B, Baillie GS (2018). FBXW7 regulates DISC1 stability via the ubiquitin-proteosome system. Mol Psychiatry.

[51] Sin YY, Martin TP, Wills L, Currie S, Baillie GS (2015). Small heat shock protein 20 (Hsp20) facilitates nuclear import of protein kinase D1 (PKD1) during cardiac hypertrophy. Cell Commun Signal..

[52] Martin TP, Hortigon-Vinagre MP, Findlay JE, Elliott C, Currie S, Baillie GS (2014). Targeted disruption of the heat shock protein 20-phosphodiesterase 4D (PDE4D) interaction protects against pathological cardiac remodelling in a mouse model of hypertrophy. FEBS Open Bio.

