# Supplementary material for: Targeting B-Raf inhibitor resistant melanoma with novel cell penetrating peptide disrupters of PDE8A – C-Raf
Source: BMC Cancer. 2019 Mar 25;19:266. doi: 10.1186/s12885-019-5489-4 (PMC6434832; doi:10.1186/s12885-019-5489-4)
Supplement: Supplementary file 1 — Table S1. Chemicals and Antibodies used in study. (DOCX 67 kb) [file 12885_2019_5489_MOESM1_ESM.docx]

**Supplementary Information**

Supplementary Table 1: Chemicals and Antibodies

| **Reagent** |  | **Source** |
| --- | --- | --- |
| PDE8A – C-Raf Disruptor Peptide  *Stearic Acid [CH_3_(CH_2_)_16_COOH] conjugated to C terminus* | RRLSGNEYVLST | GenScript |
| Scrambled Peptide  *Stearic Acid [CH_3_(CH_2_)_16_COOH] conjugated to C terminus* | SYTVRLLGERNS | GenScript |
| Anti-phospho-ERK rabbit primary antibody | 1:1000 | Sigma-Aldrich |
| Anti-phospho-ERK1/2 (Y204/T202) rabbit primary antibody | 1:1000 | Cell Signalling |
| Anti-GAPDH mouse primary antibody | 1:2000 | Santa Cruz |
| Donkey anti-mouse LI-COR 700nm secondary antibody | 1:5000 | LI-COR |
| Donkey anti-rabbit LI-COR 800nm secondary antibody | 1:5000 | LI-COR |
| PLX4032 (Vemurafenib, RG7204) | Final conc.  [1µM] | Sellekchem |
