# Supplementary material for: Targeting B-Raf inhibitor resistant melanoma with novel cell penetrating peptide disrupters of PDE8A – C-Raf
Source: BMC Cancer. 2019 Mar 25;19:266. doi: 10.1186/s12885-019-5489-4 (PMC6434832; doi:10.1186/s12885-019-5489-4)
Supplement: Supplementary file 2 — A375 growth data. Single-treatment of 10 μM PPL-008C and PPL-008CSS significantly attenuated A375 cell (V700E malignant melanoma cell line) growth although pERK levels are unaffected by these treatments (see Fig. 1.). (PPTX 118 kb) [file 12885_2019_5489_MOESM2_ESM.pptx]

## Slide 1
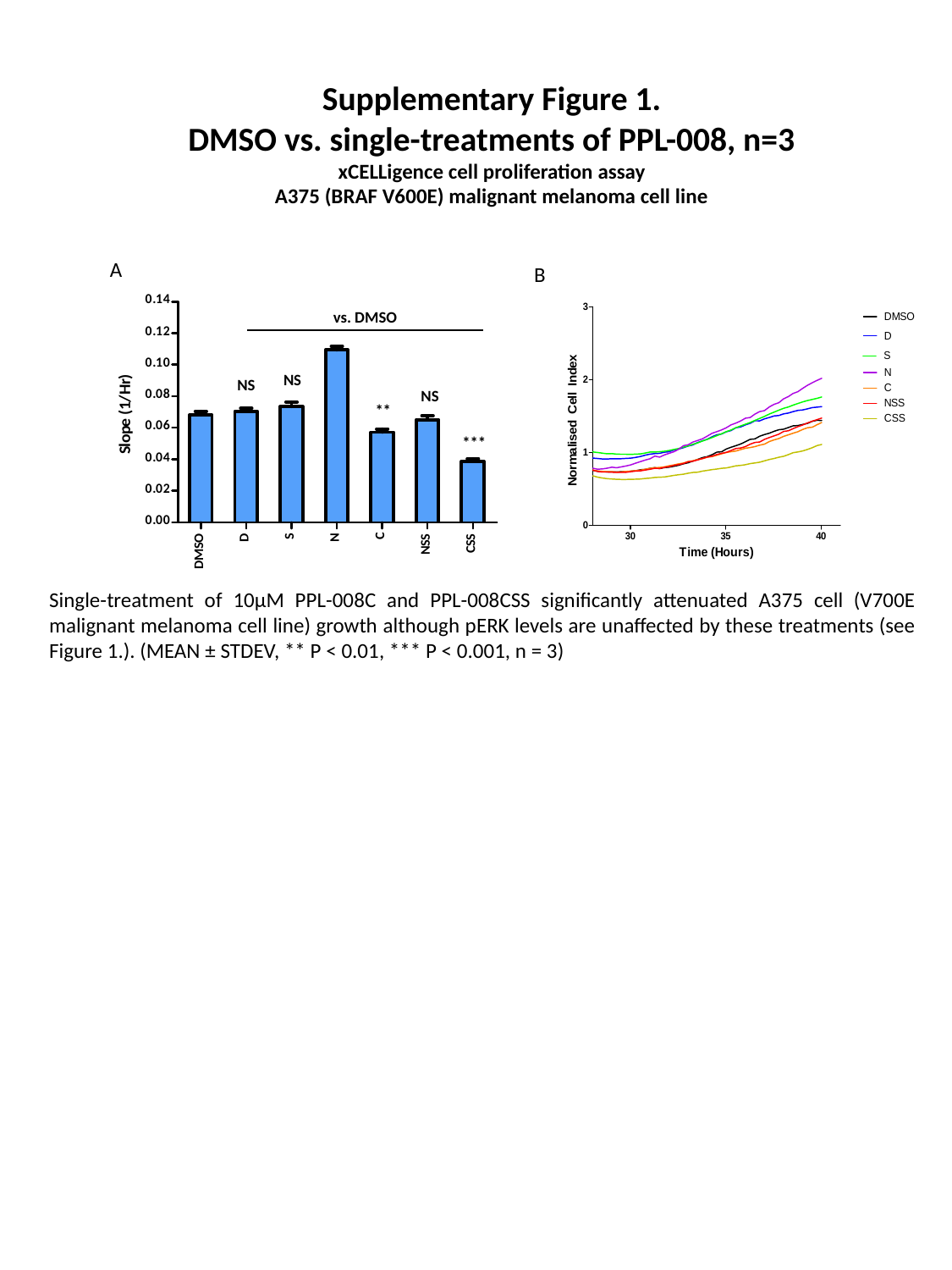

Supplementary Figure 1.
DMSO vs. single-treatments of PPL-008, n=3
xCELLigence cell proliferation assay
A375 (BRAF V600E) malignant melanoma cell line
A
B
vs. DMSO
NS
NS
NS
**
***
Single-treatment of 10µM PPL-008C and PPL-008CSS significantly attenuated A375 cell (V700E malignant melanoma cell line) growth although pERK levels are unaffected by these treatments (see Figure 1.). (MEAN ± STDEV, ** P < 0.01, *** P < 0.001, n = 3)
